# Animal models for understanding the mechanisms of malnutrition: a literature review

**DOI:** 10.3389/fnut.2025.1655811

**Published:** 2025-09-25

**Authors:** Muhammad Shahzad, Habab Ali Ahmad, Mustajab Ghani, Ziad Al Nabhani

**Affiliations:** ^1^Faculty of Dentistry, Zarqa University, Zarqa, Jordan; ^2^Institute of Basic Medical Sciences, Khyber Medical University, Peshawar, Pakistan; ^3^Department of Biological and Health Sciences, Pak-Austria Fachhochschule Institute of Applied Sciences and Technology, Haripur, Pakistan; ^4^Institute of Health Sciences, Khyber Medical University, Swat, Pakistan; ^5^Department of Visceral Surgery and Medicine, Bern University Hospital, Bern, Switzerland; ^6^Maurice Müller Laboratories, Department for Biomedical Research, University of Bern, Bern, Switzerland

**Keywords:** malnutrition mechanism, rodent model, non-human primates, undernutrition, pigs, zebrafish

## Abstract

Malnutrition, encompassing undernutrition, micronutrient deficiencies, and overnutrition, remain a pervasive global health challenge. This underprivileged condition contributes significantly to worldwide morbidity and mortality and causes profound impairments in growth, development, immune function, and metabolic health. Understanding the underlying biological mechanisms is critical, and animal models are indispensable tools for dissecting these complex pathways and for evaluating potential nutritional interventions under controlled conditions that are infeasible in humans. This literature review comprehensively examines rodent models and explores other diverse animal models used to investigate malnutrition, ranging from invertebrates (e.g., Drosophila) and fish (zebrafish) to mammals (piglets and non-human primates). We highlight how each model has yielded mechanistic insights into malnutrition-induced pathophysiology, i.e., from altered metabolic signaling to immune dysfunction and critically evaluate their strengths and limitations in replicating the multifactorial nature of human malnutrition. Key considerations include the extent to which each model mimics human nutritional deficits or excesses, appropriate developmental stages, species-specific metabolic differences, and the influence of comorbid factors such as infection or gut microbiome alterations. We emphasize translational relevance by identifying where animal-derived findings align with clinical observations and where they diverge, underscoring the challenges in extrapolating preclinical results to human disease. Overall, this review provides a comprehensive resource to guide researchers in selecting appropriate animal models and interpreting their findings, with the ultimate goal of enhancing the translation of preclinical insights into improved strategies to address malnutrition.

## 1 Introduction

Malnutrition, encompassing both undernutrition and over nutrition, stands as a prevalent global health challenge. Understanding the underlying mechanisms of malnutrition is of paramount importance because it lays the foundation for the creation of interventions that are not only effective but also precisely targeted. In this context, animal models-based research serves as indispensable tools to unravel underlying mechanisms involved in malnutrition. Animal models allow researchers to investigate complex physiological processes, metabolic pathways, and long-term effects of nutritional deficiencies or excesses in controlled environments. These models enable the study of specific nutrient interactions, gene expression changes, and systemic responses to malnutrition that would be challenging or unethical to examine in human subjects. By replicating various forms of malnutrition in animals, scientists can elucidate the underlying molecular and cellular mechanisms, identify potential biomarkers, and test novel therapeutic approaches. Furthermore, animal studies provide valuable insights into the developmental consequences of malnutrition, particularly during critical growth periods, which can inform strategies for preventing and mitigating long-term health impacts on humans. However, it is essential to acknowledge the inherent limitations of animal models. The physiological differences between animals and humans, as highlighted in the literature, necessitate careful interpretation and extrapolation of findings. It is currently accepted that several factors such as species-specific metabolic rates, digestive systems, and immune responses, can influence the manifestation and progression of malnutrition. Therefore, a critical evaluation of each model’s relevance to human conditions is paramount.

This review provides a comprehensive exploration of animal models that are currently employed in the study of malnutrition, with a specific focus on highlighting their individual strengths, limitations and translational potential offering a unique resource over existing literature by integrating recent omics and microbiome insights to guide evidence-based interventions for global health challenges.

## 2 Malnutrition overview

### 2.1 Malnutrition definition and types

Malnutrition refers to the deficiencies or excesses in nutrient intake, an imbalance of essential nutrients, or impaired nutrient utilization. Over the last two decades, the definition of malnutrition has gradually evolved leaving a significant impact on advancing the understanding of malnutrition at both academic and clinical levels (1). Generally, the definition of malnutrition is limited to the deficiencies, excesses, or imbalances of energy and/or nutrients of an individual (2). While *The Lancet* has covered various aspects of malnutrition at length, the comprehensive definition from the World Health Organization (WHO) addresses both undernutrition and over nutrition (2). WHO categorize malnutrition into three broad categories: (a) undernutrition including stunting, wasting and underweight (b) micronutrients malnutrition encompassing deficiency or excess of one or more micronutrients such as vitamins and minerals, and (c) overweight including obesity and diet-related, non-communicable diseases (such as heart disease, stroke, diabetes, and some cancers) (3). Throughout this review, ‘malnutrition’ serves as the overarching term encompassing undernutrition (e.g., stunting, wasting), over nutrition (e.g., obesity), and micronutrient imbalances, with specific sub-terms used for precision.

### 2.2 Global burden of malnutrition

Malnutrition is still a global issue of public health concerns despite a gradual decline in prevalence over the last three decades. According to the latest WHO estimates, around 2.5 billion adults across the world were overweight and 390 million were underweight in 2022. Malnutrition was also common in children under 5 years of age with approximately 149 million stunted, 45 million wasted and 37 million overweight or obese children globally, during the same period. Malnutrition consequences are devastating, especially in children with around 45 million under five affected by wasting worldwide (4). The burden of malnutrition is greatest in low and middle-income countries with South Asia and Sub-Saharan Africa being the hardest hit region of the world. It is estimated that around two-thirds of the 150.2 million stunted children reside in these two regions only (5). More recently, low-income and middle-income countries are faced with a rising problem of the double burden of malnutrition. The phenomena, characterized by the coexistence of under and overnutrition, at the same time, affect one third of all LMICs posing significant threat to the health and well-being of the masses. Another closely associated problem is the adverse impact of recent poly-crises (existence of more than one major crises at the same time such as war, climate change, pandemic, inflation and food insecurity) on chronic malnutrition which will likely further exacerbate the existing situation (6). Successful tackling of malnutrition problem require evidence based, cost effective, and sustainable interventions.

### 2.3 Impact of malnutrition on health

Malnutrition has a significant impact human health at every stage of life. During early life, malnutrition contributes to nearly 45% deaths in children under five years of age, largely by weakening immune defenses and increasing vulnerability to infections (7). Beyond immediate mortality risk, malnutrition in early life has severe short-term effects on growth, development, and overall health and wellbeing. A child who is malnourished experiences impaired physical growth (manifesting as low weight-for-age and height-for-age), recurrent illnesses due to immune suppression, and delayed cognitive and motor development (8). For example, undernourished children exhibit higher rates of developmental delay and infections such as pneumonia and diarrheal disease. If acute malnutrition is not treated on time, these early insults can become chronic, leading to **s**tunting, a state of irreversible growth failure associated with long-term deficits in cognition and school performance. Indeed, poor nutrition during the first 1,000 days of life (from conception through toddlerhood) can cause permanent reductions in a child’s cognitive ability and educational attainment. A recent systematic review confirmed that childhood undernutrition is strongly associated with impaired neurodevelopment, lower IQ and academic achievement, and behavioral problems (9). In turn, these deficits translate to diminished productivity and economic potential in adulthood (10). In adolescents and adults, chronic undernutrition leads to muscle wasting, weakness, and fatigue, which reduces work capacity and quality of life. The immune system is compromised at all ages, making malnourished individuals of any age more susceptible to diseases such as tuberculosis and other infections (11). Pregnant women who are malnourished are more likely to give birth to low-birth-weight infants, perpetuating an intergenerational cycle of poor health. The long-term consequences of early-life malnutrition extend into adulthood in myriad ways. Childhood stunting has “long-lasting physiologic effects” and is associated with increased risks of adult-onset conditions such as obesity, type 2 diabetes, and cardiovascular disease (12). These findings support the concept of developmental programming, whereby early nutritional deprivation permanently alters metabolism and organ function. For example, survivors of severe famine in utero or early childhood have shown higher rates of hypertension, glucose intolerance, and other metabolic disorders in mid-life (13). Such outcomes are thought to arise when a body conditioned to scarcity (undernutrition) is later exposed to energy sufficiency or excess, leading to a mismatch that overwhelms homeostatic capacity.

Importantly, malnutrition involves not only macronutrient deprivation (protein-calorie undernutrition) but often multiple micronutrient deficiencies. Lacking essential vitamins and minerals produces classic syndromes that compound health problems. Iron deficiency, for instance, causes anemia and fatigue, while vitamin A deficiency leads to night blindness and impairs immunity, increasing infection risk. In malnourished children, deficiencies of zinc and vitamin D contribute to poor immune function, stunted bone growth (rickets in children), and greater infection severity (14–16). Iodine deficiency in pregnancy can result in cretinism – severe, irreversible neurodevelopmental impairment in the child, underscoring how specific nutrient deficits during critical periods devastate human capital (17). These insights emphasize that malnutrition’s effects span from molecular deficits (vitamins, minerals) to whole-body dysfunction, affecting virtually every organ system. In summary, malnutrition in early life impairs growth, immune defense, and cognitive development in the short term, and sets the stage for chronic health issues and socio-economic disadvantages in the long term.

## 3 Animal models in nutrition research

### 3.1 Historical context of animal models in nutritional science

The use of animal models in nutrition research dates back to the early 19th century when François Magendie, in 1816, demonstrated that dogs fed on a diet devoid of protein (nitrogen) could not sustain life thus establishing the essential role of dietary proteins in health (18). By the late 19th century, animal model-based research played a pivotal role in identifying certain unknown dietary factors later known as vitamins for their crucial role in health and disease. For example, Christiaan Eijkman, in 1890, observed that chickens fed only polished rice developed neuropathy (resembling human beriberi), which was cured by restoring rice bran, leading to the discovery of thiamine (vitamin B₁) (19). In 1907, Axel Holst and Theodor Frölich induced scurvy in guinea pigs, one of the few animals like humans, that require dietary vitamin C, thereby validating a model for vitamin C deficiency (20). Similarly, dogs were used in the 1920s to identify niacin as the missing nutrient in pellagra (“black tongue” in dogs) (21). Rats quickly became a mainstay of early 20th-century nutrition science, leading to the discovery of fat-soluble and water-soluble vitamins by carefully controlled feeding studies (22). These foundational experiments established animal models as indispensable tools in defining malnutrition not just as caloric deprivation but as specific nutrient deficiencies.

### 3.2 Rationale for using animal models in malnutrition studies

Animal models remain crucial in malnutrition research because they allow controlled mechanistic investigations that would be impossible or unethical in humans. Current knowledge about nutrient functions, requirements, and interactions is based largely on animal studies under defined diets. In contrast to human studies, animal experiments can isolate one variable (e.g., protein intake or a single micronutrient) and rigorously test its effect on physiology, thereby revealing causal relationships (23). For example, experimental models have been used to demonstrate how protein or micronutrient deficiencies impair immune development and host defense, or how early-life undernutrition can program long-term metabolic outcomes (24, 25). With animals, researchers can perform frequent sampling of tissues and observe effects on organ systems (brain, liver, immune organs, etc.) across the lifespan or even across generations in a relatively short time. Such studies have provided mechanistic insight into the links between malnutrition and outcomes like stunted growth, cognitive deficits, and altered immunity. Another key rationale is translational research: animal models serve as a bridge to human applications. Promising nutritional interventions (therapeutic diets, micronutrient supplements, microbiome therapies, etc.) can be first tested for efficacy and safety in animals (26). Indeed, many therapeutic foods and supplement strategies were optimized in animal trials before being applied clinically. The ability to control experimental conditions tightly (genetics, environment, diet composition) in animal models yields high-quality data that, with careful interpretation, can guide human studies (27). In sum, animal models offer a level of experimental precision and the opportunity for invasive analysis (e.g., organ histology, gene expression) that together provide invaluable insight into the mechanisms of malnutrition and potential interventions.

### 3.3 Ethical considerations and regulatory frameworks

The use of animals in malnutrition research carries important ethical obligations. Researchers must ensure that studies are justified and humane, adhering to the 3Rs principle: Replacement, Reduction, Refinement. *Replacement* means using non-animal alternatives whenever possible, for instance, cell cultures or computer models, although for integrated whole-body nutritional effects, animal models are often still necessary. *Reduction* refers to using the minimum number of animals required to achieve scientific objectives, employing good experimental design and statistical power analysis to avoid waste. *Refinement* involves minimizing pain and distress for the animals: in malnutrition experiments, this can include careful monitoring of body condition, setting humane endpoints (e.g., stopping a dietary restriction if an animal loses a certain percentage of body weight or shows signs of severe illness), and providing supportive care as appropriate. Since severe nutrient deprivation can cause suffering, researchers must balance scientific aims with animal welfare, perhaps by inducing milder degrees of malnutrition or shorter durations if that suffices to model the condition. These 3Rs principles, first proposed by Russell and Burch in 1959, have become enshrined in legislation and guidelines governing animal research (28). Institutional Animal Care and Use Committees (IACUCs) or ethics boards oversee malnutrition studies to ensure compliance with welfare standards and that no feasible alternative exists. Species choice is also an ethical consideration: scientists preferentially use less sentient or simpler organisms (e.g., rodents or fish) in place of higher primates unless the research question absolutely requires a close human analog (29). For example, while a macaque model might yield unique data on cognitive development under malnutrition, a rodent is typically used for initial studies, given the much lower ethical and financial burden. All animal experiments are conducted under licenses or regulations that demand humane housing, feeding, and handling. In practice, implementing ethical frameworks not only protects animals but also improves science: refined techniques and healthy, unstressed animals yield more reliable, interpretable results. In summary, the scientific rationale for animal models must always be weighed against ethical imperatives, and researchers are duty-bound to design malnutrition studies that maximize knowledge gained while minimizing harm to animals.

## 4 Commonly used animal models for malnutrition research

**Figure 1:**
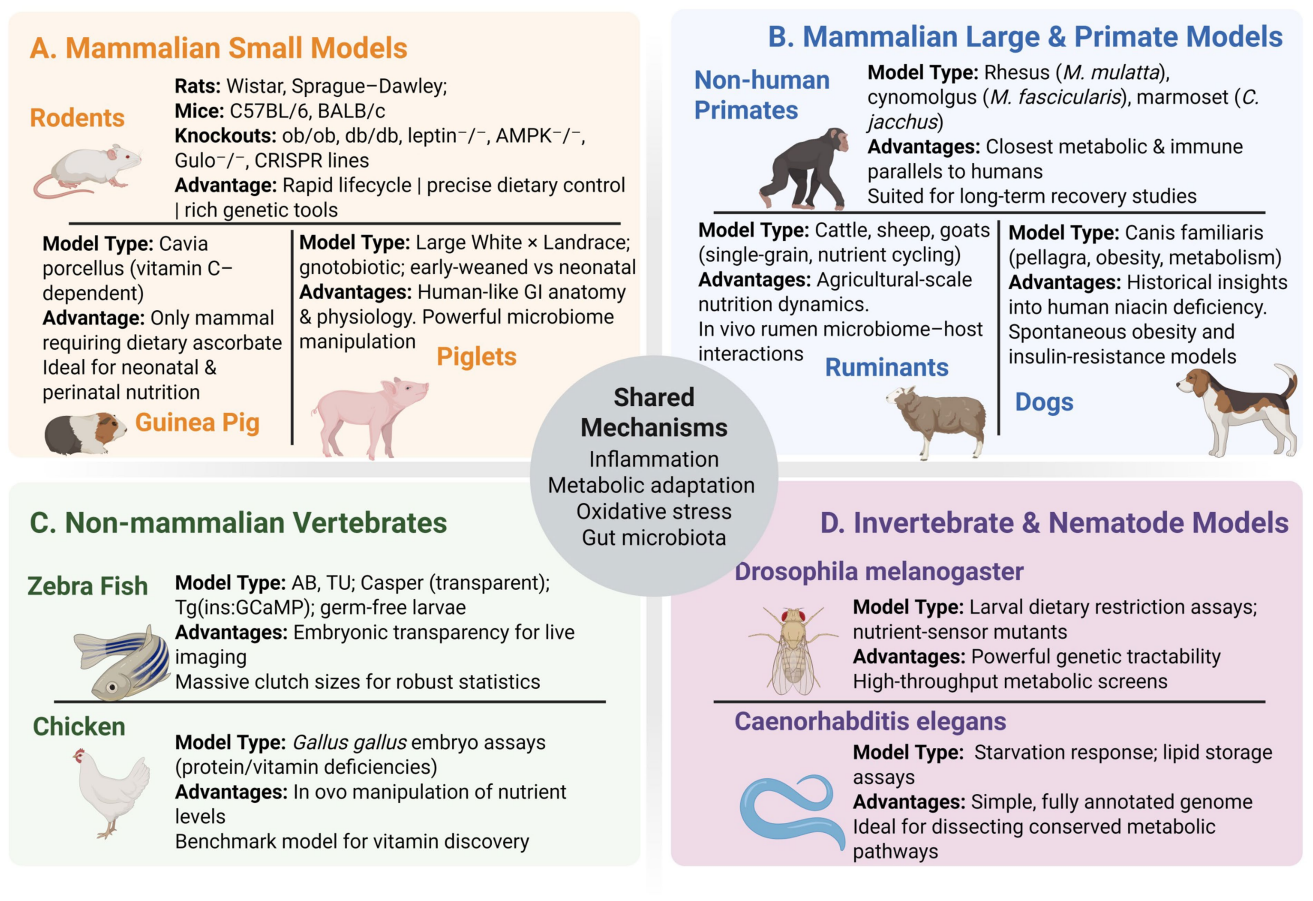
Comprehensive overview of animal models used to study malnutrition, organized by taxonomic group. Mammalian small models **(A)** include rodents (Wistar, Sprague–Dawley, C57BL/6, BALB/c, and key knock-out lines), guinea pigs (ascorbate-dependent *Cavia porcellus*), and piglets (conventional, gnotobiotic, early-weaned vs. neonatal), each valued for rapid lifecycles, ascorbate dependency, or human-like gut physiology. Mammalian large and primate models **(B)** comprise non-human primates (rhesus, cynomolgus, marmoset), dogs (pellagra/obesity models), and ruminants (cattle, sheep, goats), chosen for their translational relevance, historical insights, or rumen microbiome dynamics. Non-mammalian vertebrates **(C)** cover zebrafish strains (AB, TU, Casper, reporter lines, germ-free larvae) and chicken (*Gallus gallus* embryo assays), offering live imaging and in-vivo nutrient manipulation. Invertebrate and nematode models **(D)** include *Drosophila melanogaster* (dietary restriction assays, nutrient-sensor mutants) and *Caenorhabditis elegans* (starvation response, lipid storage assays) for high-throughput genetics and conserved metabolic pathway studies. The central hub highlights shared mechanisms: inflammation, metabolic adaptation, oxidative stress, and gut microbiota.

A wide range of animal species have been employed to model malnutrition, each offering unique advantages for probing specific aspects of undernutrition, overnutrition, or micronutrient deficiencies. Figure 1 provides an overview of commonly used models, spanning rodents to non-mammalian species, along with their key features and research applications. No single model recapitulates human malnutrition in its entirety; instead, each species contributes complementary insights. Below, we discuss how each model has advanced understanding of malnutrition’s effects and identify species-specific strengths and limitations.

### 4.1 Rodent models (mice and rats)

Mice (*Mus musculus*) and rats (*Rattus norvegicus*) are the most frequently used laboratory models in nutrition research. Their small size, short reproductive cycles, and relatively low cost allow for large study cohorts and even multigenerational experiments. A particularly powerful feature of mice is their genetic tractability, i-e the availability of inbred strains and modern genomic editing (transgenics, knockouts) enables researchers to dissect gene-nutrient interactions with precision. Rodents share a high degree of physiological and genetic homology with humans, which permits reasonable extrapolation of findings in many cases. For example, mice and rats have been used to elucidate mechanisms of protein-energy malnutrition (PEM) by feeding low-protein or calorie-restricted diets that produce weight loss, stunting, muscle wasting, and immune dysfunction analogous to human (26, 30–32). Such models have confirmed causative links between inadequate protein intake and outcomes like impaired glucose homeostasis and loss of lean body mass. Rodent models have also been central to micronutrient deficiency research: iron-deficient diets in rats (33) induce anemia and cognitive impairment, zinc deficiency in mice (34) leads to growth failure and immune deficits, and vitamin A deficiency (35) in rodents causes vision problems and susceptibility to infection. Notably, many vitamin requirements for humans were first identified by studies in rats. Rodents have further proven invaluable in studying overnutrition and diet-related diseases; high-fat or high-sugar diets in mice can induce obesity, type 2 diabetes, and nonalcoholic fatty liver disease, shedding light on metabolic syndrome under conditions of food excess. Neonatal rodent models (especially mice) are used to mimic early-life malnutrition and have demonstrated how inadequate nutrition during critical windows can disrupt organ development (e.g., brain growth, immune system maturation) and even program metabolic changes that persist into adulthood. Such developmental programming studies in rodents underpin the DOHaD (Developmental Origins of Health and Disease) concept (36). Transgenerational effects have also been examined, for instance, malnourishing pregnant or lactating rodents can produce offspring with long-term deficits, helping to parse how maternal nutrition influences the next generation. Overall, rodent models have contributed to virtually every domain of malnutrition research, from basic nutrient physiology to testing of interventions (such as fortified diets or gut microbiome therapies) in a controlled setting. Table 1 summarizes several mouse models of malnutrition and their characteristics.

**Table 1:** Overview of experimentally induced malnutrition and metabolic–immune challenge models in rodents.

Animal model	Species	Induction method/Diet	Key features/Malnutrition type	Advantages	Limitations	References
GM15 Gnotobiotic model	C57BL/6 J Mice	Germ-free derivation + colonization with a 15-member defined microbiota consortium (e.g., Clostridium sp., *Bacteroides acidifaciens*, *Lactobacillus johnsonii*) Isocaloric status: Optional depleted diet (4% protein, 2% lipids) not isocaloric; maintenance diet quasi-isocaloric with carb compensation.	Mimics enteropathy and undernutrition; microbiota-driven effects; recapitulates SOPF/SPF phenotypes	Controls microbiota variables; reproducible across facilities; stable colonization over generations	Requires germ-free facilities; complex setup; limited microbial diversity compared to natural microbiota	Darnaud et al. (88)
Wistar rat undernutrition model	Wistar rats	Custom diet formulations (specific composition not detailed but designed to mimic undernutrition). Two groups Well Nourished, WN = 14% protein diet Under Nourished, UN = 5% protein diet Isocaloric status: Diets not isocaloric (UN hypocaloric at 38 kcal/day vs. WN 60.2 kcal/day; no compensation for reduced protein)	Evaluates drug efficacy in undernutrition	Cost-effective for preclinical studies	Limited data on exact diet composition; potential variability across studies	Merino Sanjuán et al. (89)
EFA-deficient rat models	Rats	Low-protein diet containing 5% casein was administered for 14–21 days to induce malnutrition	Reduced body weight, hypoalbuminemia, and muscle wasting, which are hallmarks of severe childhood malnutrition	Simple dietary intervention; rapid induction of malnutrition	Short-term focus; limited applicability to chronic malnutrition studies	Hart et al. (90)
Genetic obesity models	Mice/Rats	Monogenic mutations (e.g., *db/db*,*ob/ob*, Zucker fatty, OLETF rats) Zucker fatty rats (fa/fa) Leptin-deficient (ob/ob) mice Leptin receptor-deficient (db/db) mice	Mimics obesity and metabolic syndrome; hyperphagia, insulin resistance, hyperlipidemia	Well-characterized phenotypes	Monogenic vs. polygenic human obesity Limited applicability to polygenic human obesity; lacks complexity of environmental factors	Aleixandre and Miguel (91), Drel et al. (92), and Paeschke et al. (93)
Protein malnutrition with enteropathy	Mice	Protein-deficient diet for 5–28 days Low-protein diet (7% protein) for 3 weeks; optionally combined with indomethacin or lipopolysaccharides (LPS) Isocaloric status: Low-protein diet isocaloric to control (3.395 kcal/g; carbs increased for protein reduction)	Stunting, wasting, intestinal inflammation, gut hyperpermeability; mimics environmental enteric dysfunction (EED)	Combines undernutrition and gut pathology; recapitulates key features of human EED	Requires prolonged dietary intervention; complex setup with additional triggers (e.g., LPS, indomethacin)	Salameh et al. (25) and Salameh et al. (94)
Complementary feeding phase model	Mice/ C57BL/6 Mice	Diet mimicking undernourished children during weaning. MAL-ED diet (moderate deficiency in energy, protein, lipids, and zinc; moderate increase in carbohydrates and fiber) based on complementary feeding of undernourished children Isocaloric status: MAL-ED diet hypocaloric (−8% energy vs. control; partial carb/fiber compensation for reduced protein/lipids)	Weight loss, reduced body energy reserves, blunting of villus area, increased intestinal permeability; mimics undernutrition during complementary feeding	First murine model specific to the complementary feeding phase; clinically relevant to undernourished children	Requires precise dietary formulation; limited analysis of microbiota or biomarker	Ribeiro et al. (95)
Restricted diet-induced malnutrition	Mice/BALB/c Mice	Caloric restriction (12% weight loss) Specifically: Hypocaloric/hypoprotein/hypolipidic diet (36.26% carbohydrate, 8.79% protein, 4.95% fat) for 16 days Isocaloric Status: Restricted diet hypocaloric (7.62 kJ/100 g vs. 15.24 kJ/100 g control; no compensation)	Weight loss (12%), reduced body mass index (BMI), leukocyte count reduction (47.5%), cholesterol increase (2-fold); mimics marasmic malnutrition	Mild, reproducible malnutrition with reversible features; mimics real-world food scarcity	Short-term focus limits applicability to chronic malnutrition studies; may not fully represent prolonged food insecurity	Ferreira-Paes et al. (24)
Protein-energy malnutrition (PEM)	Wistar rats	Diet: 5–10% protein diet for 21 days Specific Diet: Low-protein diet (10% protein) vs. normal diet (18% protein) for 40 weeks Isocaloric Status: Low-protein diet isocaloric status unknown	Reduced body weight, hypoalbuminemia, hepatic steatosis, altered biochemical markers (e.g., AST, ALT, ALP); mimics chronic malnutrition	Long-term study design; evaluates physiological, hematological, biochemical, and histological changes	Requires prolonged dietary intervention; limited to post-weaning phase	Augustin et al. (37)
Zinc-deficient model	C57BL/6 J Mice	Diet: Zinc-deficient diet (30 μmol/kg) for 28 days Pair-fed controls used. Zinc-deficient diet (1 ppm zinc) + deionized distilled water for 10 weeks Isocaloric Status: Zinc-deficient diet likely isocaloric (only zinc reduced; no macro changes detailed)	Growth retardation, dermatitis, immune dysfunction; weight loss observed after 28 days of zinc-deficient diet (ineffective at 14 days)	Cost-effective and reproducible dietary approach; validated plasma and urine zinc quantification	Requires specialized diets; strain-dependent responses.	Wenegieme et al. (96)
AMPK liver-specific deletion	Mice (BALB/c or C57BL/6 J)	Diet: Low-protein (8%), high-carbohydrate diet + liver-specific AMPK deletion Isocaloric status: Low-protein diet likely isocaloric (high-carb compensation; energy not specified)	Reduced body weight, impaired fatty acid oxidation, hepatic steatosis; mimics metabolic adaptation to protein deficiency	Links genetics to metabolic adaptation; mimics real-world malnutrition scenarios	Requires genetic engineering; limited to protein-energy studies.	(97)
Maternal deprivation model	Wester Rats	Method: Maternal Diet: 50% food restriction during the last third of pregnancy (days 14–21). The Control group dams fed ad libitum Isocaloric Status: Low-protein diet not isocaloric (hypocaloric; no compensation for 4% protein vs. 20% control)	Malnutrition type: Intrauterine growth restriction (IUGR). Key Features: Low birth weight (18.3% reduction). Rapid catch-up growth, hyperphagia (61% ↑ milk intake). Obesity at weaning (18.8% ↑ body weight). Metabolic syndrome: hyperglycemia, hypertriglyceridemia, insulin resistance (TyG index ↑6.9%). Altered milk composition (↑ glucose, cholesterol, triglycerides, ghrelin; ↓ leptin, corticosterone).	Controlled experimental conditions. Mimics human intrauterine malnutrition (e.g., famine). Highlights critical window (late gestation) for metabolic programming. Demonstrates early-onset obesity and hypothalamic GHS-R upregulation.	Species-specific (rat vs. human biology). Limited to male offspring analysis. Short-term outcomes (weaning age); lacks adult data. Strain-dependent (Wistar-specific responses).	de Souza Parrela et al. (98)
Germ-Free + pathogenic colonization	C57BL/6 J, BALB/c, or other strains	Method: Colonization: Introduction of specific pathogens (e.g., *Salmonella enterica* Typhimurium, *Citrobacter rodentium*, Clostridioides difficile, *Listeria monocytogenes*) via oral gavage, intragastric inoculation, or co-housing. Isocaloric Status:	Pathogen-Driven Outcomes Increased susceptibility to infections (e.g., Salmonella causes severe colitis; *C. difficile* induces antibiotic-associated diarrhea). Immune dysregulation/ Impaired Th17 responses, reduced regulatory T cells (Tregs), and defective IgA production in GF mice. Disease models: Colitis (e.g., *C. rodentium* triggers attaching/effacing lesions). Sepsis (e.g., Listeria exploits impaired innate immunity). Colorectal cancer (e.g., *Fusobacterium nucleatum* exacerbates tumorigenesis).	Enables study of host-pathogen interactions without microbiota interference. Reveals roles of specific pathogens in immune activation or disease progression. Evaluates vaccines/antibiotics in a simplified system	Lacks complex microbiota interactions, differnce across mouse strains Requires optimization. Baseline immune defects.	Jans and Vereecke (99)
High-carbohydrate diet model	C57BL/6	Diet: High-fat (58% kcal; saturated fat + trans fats), high-carbohydrate (25% kcal; 55% fructose + 45% sucrose in drinking water) for 20–30 weeks. Control: Low-fat diet (11% kcal fat) + normal water. Isocaloric Status: N/A, High fat diet (not reduced); likely hypercaloric vs. control	Obesity, insulin resistance, hyperlipidemia. Severe hepatic steatosis, inflammation, and progressive fibrosis (perisinusoidal and bridging fibrosis). Elevated ALT, AST, fasting glucose, triglycerides, and hydroxyproline. Activation of hepatic stellate cells (HSCs) and collagen deposition	Mimics human NASH with metabolic syndrome (obesity, insulin resistance). High success rate (100% fibrosis in 30-week group) and zero mortality. Suitable for studying liver fibrosis progression and drug testing.	Long induction period (20–30 weeks). Strain-specific (C57BL/6 mice prone to metabolic disorders). Requires high-fat + high-sugar combination (not purely carbohydrate-driven).	Xin et al. (100)
Neonatal fasting model	C57BL/6	Fasting protocol: 16-h staggered fasting (ZT12-ZT4) with ad libitum feeding controls. Monitoring: Indirect calorimetry to measure respiratory exchange ratio (RER), glucose/fat oxidation. Rescue Experiments: Pharmacological interventions (e.g., FGF21, CPI-613). Isocaloric status: N/A (fasting inherently hypocaloric)	Metabolic features: Shift from glucose to fatty acid oxidation during fasting (↓ RER). - Upregulated circadian genes (e.g., Per1, Pdk4). - Altered heat production and energy substrate utilization. Circadian link: Disrupted clock-gene expression under fasting (e.g., Bmal1 deficiency exacerbates metabolic defects)	Integrates fasting metabolism with circadian rhythm analysis. Uses pharmacological rescue (FGF21, CPI-613) to validate mechanisms. Non-invasive indirect calorimetry for real-time metabolic tracking.	Requires specialized equipment (PhenoMaster). Stress from fasting/single housing may confound results. Limited to short-term fasting (16 h). Neonatal-specific data not explicitly addressed in protocol.	Sun and DeBosch (101)
Maternal food restriction during late gestation	Primiparous Sprague Dawley rats	50% food restriction from gestational day 10 until birth, based on ad libitum-fed control dams’ intake; standard chow post-birth. Isocaloric Status: 50% restriction hypocaloric (no compensation)	Global undernutrition leading to intrauterine growth restriction (IUGR); reduced birth weight, catch-up growth, altered insulin sensitivity, and behavioral changes like impulsivity.	Mimics human IUGR outcomes effectively; allows study of molecular mechanisms in brain development and metabolic programming; cost-effective and reproducible in rodents.	Induces maternal stress which may confound results; primarily studied in males, limiting sex-specific insights; short-term restriction may not capture full perinatal effects.	Batra et al. (102)
Maternal caloric restriction with protein variation during pregnancy	C57Bl/6	30% caloric restriction from embryonic day 10.5 to delivery; isocaloric diets with standard (20% protein) or high (40% protein) casein content	Protein-energy malnutrition; induces fetal undernutrition, elevated maternal/fetal corticosterone, programmed hypertension, and cardiac remodeling in offspring.	Established model for cardiovascular DoHaD effects; allows dissection of protein vs. calorie effects; translatable to human undernutrition scenarios.	Potential sex bias (often male-focused); mouse-specific metabolic responses; requires further renal and long-term studies for full translation	Kawamura et al. (103)

GM15, gnotobiotic model harboring a 15-member defined microbiota; SPF, specific pathogen–free; SOPF, specific-opportunistic pathogen–free; WN, well nourished; UN, undernourished; EFA, essential fatty acid; EED, environmental enteric dysfunction; PEM, protein-energy malnutrition; NASH, non-alcoholic steatohepatitis; MAL-ED, malnutrition–enteric dysfunction complementary feeding phase diet; BMI, body mass index; AST, aspartate aminotransferase; ALT, alanine aminotransferase; ALP, alkaline phosphatase; FGF21, fibroblast growth factor 21; CPI-613, Devimistat (mitochondrial metabolism modulator); RER, respiratory exchange ratio; GHS-R, growth hormone secretagogue receptor.

Despite their utility, rodents have important limitations. They differ from humans in gut anatomy, basal metabolic rate, and lifespan, which can affect how malnutrition manifests. Crucially, standard laboratory rodents are resistant to some clinical features of severe malnutrition, such as kwashiorkor-like edema is typically not observed in rodent models, even when diets are extremely protein-deficient (37). Rodents also synthesize certain vitamins endogenously (e.g., vitamin C), so they do not naturally develop scurvy; researchers must use special strains (such as Gulo-knockout mice) or other workarounds to study vitamin C deficiency. Furthermore, rodent behavior and cognition, while informative, are not as complex as humans’, limiting their use for studying higher-order neurodevelopmental effects (where larger animals might be preferred). Additionally, notable differences exist between the rodent and human gut microbiome, which can influence malnutrition outcomes. Comparative analyses of rodent and human gut microbiomes reveal marked interspecies divergences in taxonomic composition, functional gene repertoires, and metabolite profiles, with implications for translational validity of preclinical models. Human fecal communities are typically enriched in Bacteroides, Ruminococcaceae, and Clostridiales, exhibiting a higher Firmicutes–Bacteroidetes ratio relative to murine and rat counterparts, which often harbor greater proportions of Lactobacillus and Muribaculaceae (38, 39). Metagenomic comparisons indicate that only ~4% of bacterial genes share substantial sequence identity between the two hosts, underscoring functional disparities despite overlapping genera (38). Short-chain fatty acid patterns also differ, with humans and non-human primates showing closer *β*-diversity clustering than rodents, and mice generally more similar to humans than rats. Moreover, human-derived microbial consortia engraft with variable efficiency in germ-free rodents, with GF rats more faithfully recapitulating donor Clostridiales composition than GF mice, which exhibit selective enrichment of certain Bacteroides phylotypes (40). These differences mean that findings in mice and rats must be translated to humans with caution. Nonetheless, the ease of manipulating and observing rodents under controlled dietary regimens makes them an indispensable first-line model for malnutrition research.

### 4.2 Porcine models (pigs)

Pigs (*Sus scrofa domesticus*), especially young piglets, have emerged as valuable models in nutritional research due to their close anatomical and physiological resemblance to humans. Pig gastrointestinal tracts, digestive functions, and dietary patterns (omnivorous) are more similar to humans than those of rodents. Notably, the neonatal piglet’s developmental trajectory parallels that of human infants in many aspects: newborn pigs have a precocial developmental stage with organ maturity and metabolism comparable to a human newborn, and their brain growth spurt occurs in the early postnatal period, analogous to humans (41). These similarities make piglets an excellent model for pediatric undernutrition. Researchers can use infant piglets to study conditions like infantile malnutrition, intrauterine growth restriction, or prematurity under different feeding regimens. For example, feeding piglets a protein-deficient or micronutrient-deficient diet reliably produces symptoms of growth faltering, impaired gut function, and neurodevelopmental delays, closely mirroring human infant malnutrition. One study demonstrated that protein-restricted piglets had altered gut microbiota composition and weakened intestinal barrier function, providing insight into how malnutrition contributes to the cycle of diarrhea and nutrient malabsorption in children (a phenomenon unethical to induce experimentally in human infants) (42, 43). Pigs have also been extensively used to compare enteral vs. parenteral nutrition strategies in malnourished states; because their size permits surgical catheterization and repeated sampling, neonatal piglets are ideal for testing interventions like fortified formulas, probiotics, or therapeutic foods and observing their effects on organ systems (44). The translational relevance of pig studies is high – many findings on nutrient requirements and metabolism in pigs have directly informed clinical nutrition for human babies. Beyond the infant model, growing or adult pigs (including miniature pig breeds) serve as models of metabolic syndrome and obesity, since they can develop diet-induced obesity with metabolic and cardiovascular complications that resemble those in obese humans. Like humans, pigs deposit both subcutaneous and visceral fat and can exhibit insulin resistance, making them useful for studying overnutrition and related chronic diseases (45).

The pig model’s strengths come with some practical and ethical challenges. Pigs are large and costly to house and feed, and their longer lifespan and gestation (relative to rodents) mean experiments are more time- and resource-intensive (46). Group sizes for pig studies are usually smaller, and specialized facilities are needed for their care. Ethically, pigs are intelligent mammals, raising welfare considerations; studies must ensure proper housing and minimize stress (which can otherwise affect nutritional physiology). While piglets adapt well to laboratory rearing, they require skilled care to mimic maternal nutrition (e.g., feeding with artificial sow milk replacer). Another limitation is that even pigs do not fully recapitulate human malnutrition in the context of complex social and environmental factors; for example, infections common in malnourished children are not automatically present in experimental piglets unless introduced. Nonetheless, when it comes to translational fidelity, the pig is considered one of the best models after primates. Swine studies have been instrumental in bridging the gap between rodent findings and human clinical trials, particularly for pediatric nutrition. In summary, porcine models combine physiological similarity to humans with the ability to conduct invasive and longitudinal studies, thereby greatly enhancing our understanding of malnutrition’s impact on growth, gastrointestinal health, and organ development.

### 4.3 Non-human primate models

Non-human primates (NHPs) such as rhesus macaques (*Macaca mulatta*) and baboons (*Papio* spp.) offer the closest approximation to human biology among animal models of malnutrition. Their digestive system, immune responses, endocrinology, and neurodevelopmental processes are highly similar to humans, and they are capable of complex social and behavioral interactions (47). Because of this, NHP models have provided unique insights, particularly in areas where lower animals are too dissimilar, for example, the long-term cognitive and behavioral consequences of early-life malnutrition. Researchers have conducted studies in which pregnant or lactating monkeys are fed nutrient-restricted diets to examine effects on offspring (48). Prenatal malnutrition in rhesus macaque**s** has been shown to result in infants with significant neurodevelopmental impairments and metabolic alterations (49). In one seminal study, protein-energy malnutrition imposed on pregnant rhesus monkeys led to babies that exhibited delayed neural maturation and behavioral abnormalities, highlighting the fetal origins of cognitive deficits (49, 50). Such primate studies reinforce evidence from human cohorts that maternal undernutrition can have lasting effects on progeny (the developmental origins hypothesis), while offering a controlled experimental confirmation of causality. NHPs have also been used to model postnatal malnutrition, for instance, separating infant monkeys from adequate nutrition during the nursing period has helped investigators track the impact on brain growth, immune development, and even epigenetic changes over their lifespan (51). With the ability to follow individuals for years, NHP models uniquely enable life-course studies of malnutrition. These models can span from infancy into adolescence or adulthood over years or even decades, providing longitudinal insights into malnutrition’s life-course effects, closely mirroring the extended human developmental timeline in NHPs. This has shed light on how early stunting or wasting might predispose individuals to later health problems like obesity, diabetes, or impaired immune responses. Additionally, certain aspects of malnutrition-related pathologies are better replicated in primates: for example, rhesus monkeys on an atherogenic diet can develop arterial lesions more similar to human cardiovascular disease than what occurs in rodent models (52).

Despite these advantages, NHP models are used sparingly and only for the most critical translational questions, due to ethical and practical limitations. Non-human primates are sentient, social, and often endangered animals; experiments on them are subject to stringent ethical scrutiny (46). The 3Rs principle strongly encourages using NHPs only when no other species will suffice. Their care is expensive and requires specialized primate facilities and veterinary expertise. Reproductive rates are slow (one offspring at a time, with long gestation), so sample sizes are inherently limited. Moreover, ethical frameworks typically prevent inducing severe malnutrition in primates; studies may use moderate dietary restriction rather than life-threatening undernutrition, to avoid undue harm. These factors mean that while NHP studies provide high relevance, they are not amenable to high-throughput experimentation. Another limitation is timescale: a monkey’s developmental timeline is much longer than a rodent’s, so studies can take years or decades. For example, to observe multigenerational effects (F1, F2 generations) in primates would be impractical. Nonetheless, even a small number of well-designed primate studies have been profoundly influential (53). They’ve confirmed, in a controlled way, phenomena suspected from human data, such as the link between early malnutrition and impaired cognitive function, lending weight to public health arguments for early nutrition interventions (54). In summary, non-human primates are the closest proxy to human malnutrition and fill an important niche in research, but their use is rightly limited to questions where their unique similarity is indispensable.

### 4.4 Zebrafish models

Over the past two decades, the small tropical zebrafish (*Danio rerio*) has become a novel model organism in nutritional science, including malnutrition research. Zebrafish offer several experimental advantages: they are small and inexpensive to maintain, have high fecundity (hundreds of offspring per mating), and develop rapidly (organs form within days). Uniquely, zebrafish embryos and larvae are transparent, allowing direct observation of developmental processes and organogenesis under a microscope. Researchers can easily manipulate the nutrient content of the water or feed to which larval zebrafish are exposed, making it straightforward to create models of deficiency or overnutrition. For example, zebrafish larvae raised in water lacking certain micronutrients will exhibit developmental abnormalities relevant to that deficiency, vitamin D deficiency leads to skeletal malformations and poor bone mineralization in developing zebrafish, recapitulating rickets-like features (55). Zebrafish have also been used to study lipid metabolism; diets high in cholesterol or certain fatty acids can induce vascular changes and fat deposition, providing a proxy for studying hyperlipidemia and obesity on a microscopic scale (56, 57). Notably, zebrafish can develop diet-induced obesity and metabolic perturbations when fed a high-calorie diet, including increased adiposity and insulin-resistant phenotypes (58). These models have been utilized in nutrigenomics investigations to examine how gene expression shifts in response to overnutrition or nutrient scarcity. Another area where zebrafish excel is high-throughput intervention testing, large numbers of zebrafish larvae can be arrayed in multi-well plates to screen combinations of diets or therapeutic compounds that might improve outcomes in malnourished conditions. This approach has been applied to identify nutritional supplements that enhance growth or to test probiotics that might mitigate undernutrition effects, with readouts like growth rate, enzyme expression, or bone development assessed rapidly *in vivo* (59, 60). Additionally, zebrafish have contributed to understanding transgenerational effects of malnutrition: one study showed that parental micronutrient deficiencies in zebrafish distorted DNA methylation patterns in the liver of their offspring, implicating heritable epigenetic changes similar to those observed in mammals (61, 62). Given their genetic tractability (with tools like CRISPR available), zebrafish allow researchers to knock out or modify genes of interest to see how those changes impact nutritional phenotypes, providing a powerful way to link nutrient sensing pathways with specific genes.

The zebrafish model, while innovative, has limitations stemming from its evolutionary distance from humans. As an aquatic ectotherm, the zebrafish’s metabolism and physiology differ in fundamental ways: for example, they lack lungs, have a two-chambered heart, and excrete nitrogenous waste as ammonia into water, factors that make some aspects of malnutrition (like impacts on pulmonary development or precise basal metabolic rate comparisons) hard to translate (63). Their nutrient absorption occurs directly from water as well as from food, which is unlike human oral intake. Moreover, certain diseases related to malnutrition (such as kwashiorkor’s edema or marasmus-related infections) do not manifest in fish. Thus, while zebrafish are superb for studying basic developmental biology and molecular responses to nutrient variation, researchers must be cautious in extrapolating results to human malnutrition without corroboration in mammalian models. Emphasizing the need for translational validation, zebrafish findings (e.g., on nutrient-gene interactions) must be corroborated in mammalian models like rodents or pigs before human application, due to phylogenetic differences in metabolism and epigenetics (64). In practice, zebrafish often complement, rather than replace, rodent studies: a discovery in zebrafish (e.g., a gene that is activated during nutrient stress) can be followed up in mice for validation. Despite these caveats, the zebrafish’s contribution to malnutrition research is growing, especially in the realm of nutrient-gene interactions and rapid screening. As a high-throughput system, it helps narrow down hypotheses that can later be tested in mammals. In summary, zebrafish exemplify how a non-mammalian model can significantly advance our understanding of malnutrition’s molecular underpinnings and potential interventions, even though direct translational leaps require additional confirmation in more human-like systems.

### 4.5 Other animal models and considerations

In addition to the above models, several other species have historically played important roles in malnutrition research, though their current use varies. Guinea pigs (*Cavia porcellus*) were critical in early nutrition science, as they share humans’ inability to synthesize vitamin *C. guinea* pig experiments first proved that diets lacking fresh fruits or vegetables cause scurvy (65, 66), and they remain the standard model for studying vitamin C deficiency. Guinea pigs have also been used in perinatal nutrition studies, since their placentation and gestation have some similarities to humans (they have a hemochorial placenta), making them a model for studying fetal nutrient restriction and its effects on offspring (67). However, guinea pigs are less commonly used today outside of specific contexts like scurvy or some immunological aspects of malnutrition, in part because they are more expensive and less genetically malleable than rodents. Chickens (*Gallus gallus*) and other avian species provided early evidence linking diet and disease: Eijkman’s chicken model of beriberi was one example (68), and chick growth assays were widely used in the mid-20th century to discover vitamins (such as vitamin K and folate) and minerals by omitting them from feed and observing deficiency signs. Chicks grow rapidly, which can accentuate the effects of nutrient deficits or excesses, and they have been used to study protein malnutrition as well. In modern research, chickens are mainly utilized in agricultural and veterinary nutrition, but lessons from poultry science (e.g., on the effects of protein or mineral deficiencies on skeletal development) have parallels in human biology. Dogs (*Canis lupus familiaris*) are another historically significant model: aside from Magendie’s 19th-century protein studies, dogs were used in the early 1900s to study pellagra, where a condition called “black tongue” in dogs proved analogous to pellagra and helped identify niacin/vitamin B₃ as the preventive factor (69). Dogs were also subjects in some classic protein and energy metabolism studies due to their larger size, which allowed serial blood sampling and even fistulas for digestive studies. Today, dogs and cats are rarely used for human malnutrition research (they are more common in pet nutrition or as models for diseases like obesity and diabetes), in part due to ethical reasons and the availability of other models. Ruminants (cattle, sheep, goats) have generally been used to address agricultural malnutrition (e.g., poor pasture leading to protein-energy malnutrition in livestock) and to understand basic nutrient cycles, rather than as models for human malnutrition, their specialized digestive systems (foregut fermentation) make their metabolism quite different from humans. One notable historical example is the single-grain experiment in cattle by Stephen Babcock in 1911, which showed that cows fed only corn vs. only wheat had markedly different health outcomes, foreshadowing the discovery of micronutrients. While such large-animal work informed nutrition science, ruminants are not commonly used to emulate human malnutrition per se (70, 71).

Finally, it is worth mentioning that small invertebrate models have contributed to the fundamental understanding of nutrient sensing and energy balance. The fruit fly *Drosophila melanogaster* and the nematode *C. elegans*, for instance, have been used to unravel genetic pathways of hunger, fat storage, and metabolic adaptation to undernutrition (72, 73). These organisms offer unparalleled genetic tools and low-cost, high-throughput experimentation, moreover researchers have evolved populations of flies under chronic malnutrition to study adaptive genetic changes (74), and identified nutrient-sensing hormones and neural circuits that often have mammalian counterparts. However, due to their very simple body plans and major physiological differences, invertebrates serve as discovery engines for molecular mechanisms rather than direct models of human malnutrition. Any findings in flies or worms typically require validation in vertebrate models.

In summary, the landscape of animal models for malnutrition research is diverse. Each species, from mouse to monkey to zebrafish, brings a unique lens through which to investigate the complex problem of malnutrition. Researchers choose a model based on the specific question: mice for detailed mechanism and genetics, pigs for translational gut and infant studies, zebrafish for developmental or genomic screening, and so on. By integrating knowledge across these models, the field gains a more complete picture of how nutrient deficiencies or excesses impact living systems. Importantly, scientists remain aware of species-specific limitations and strive to cross-validate important findings in multiple models, as well as in human observational or clinical studies, to bridge any translational gaps. The table below (Table 2) summarizes key animal models, their special features, and examples of how they contribute to malnutrition research.

**Table 2:** Comparative overview of animal species employed in nutritional and malnutrition research.

Animal model	Unique features/Advantages	Primary research applications	Epigenetic mechanisms in malnutrition	Limitations
Mouse (*Mus musculus*)	Small size, short lifespan; well-characterized genome; extensive genetic tools (transgenic and knockout models); high reproductive rate	Mechanistic studies of malnutrition (cellular and molecular) Protein-energy malnutrition and cachexia models Diet-induced obesity and metabolic syndrome investigations Micronutrient deficiency effects (e.g., iron, zinc, vitamin A)	DNA methylation changes (e.g., at Agouti locus from maternal diet); histone modifications linking early nutrition to adult disease; transgenerational effects via one-carbon metabolism (folate/choline). Vulnerable in early development.	High similarity to rats in DNA methylation responses but differs from non-mammals (e.g., no placental transfer like in zebrafish); limitations: short lifespan limits long-term studies; epigenetic changes may not fully mimic human polygenic traits; ethical concerns in genetic manipulations.
Rat (*Rattus norvegicus*)	Larger rodent enabling serial sampling; historically used in nutrition discovery; intelligent with complex behavior; many inbred strains available	Developmental programming (effects of maternal/early undernutrition on later health) Classical model for vitamin and mineral deficiencies (growth and bioassay studies) Protein-calorie malnutrition models (marasmic features) Neurobehavioral studies of malnutrition (cognitive/learning tests) Chronic diet-induced disease models (e.g., high-fat diet for insulin resistance)	Maternal protein restriction causes DNA hypomethylation (e.g., in Pparα, Glut4. GR gene); histone acetylation in brain regions affecting insulin resistance and anxiety; transgenerational via steroid receptor genes.	Similar to mice in conserved methylation pathways but better for behavioral epigenetics due to complex social traits (differs from simpler models like zebrafish); limitations: less genetic tools than mice; epigenetic effects strain-dependent; may overestimate neurobehavioral impacts vs. humans.
Pig (*Sus scrofa*) – especially neonatal piglet	Omnivorous with human-like digestion and metabolism; similar gut structure and microbiota; rapid early growth akin to human infant; organs large enough for invasive monitoring	Pharmacological testing of nutritional interventions (due to easier dosing/blood collection) Infant and pediatric malnutrition models (growth faltering, developmental deficits) Gut microbiome and malnutrition interactions Parenteral vs. enteral nutrition studies in undernourished states Absorption and first-pass metabolism of nutrients/therapeutics	Low-protein diets cause DNA hypomethylation (e.g., HMGCR for cholesterol); histone mods (H3K27me3/H3K4me3) in gluconeogenesis genes; transgenerational via maternal diet affecting offspring metabolism.	Similar to rodents in methylation responses to protein restriction but more human-like gut (differs from non-mammals in microbiota-epigenome links); limitations: costly/ethical issues; epigenetic studies limited to production traits; sex-specific effects (males more affected).
Non-human primate (e.g., *Rhesus macaque, baboon*)	Closest physiological and genetic proximity to humans; long lifespan allows life-course studies; complex social behavior and cognitive development	Metabolic syndrome and obesity (in adolescent or minipig models) Prenatal malnutrition and fetal programming of disease (maternal undernutrition effects on offspring) Neurodevelopment and cognition under malnutrition (behavioral and learning outcomes) Multi-generational and longitudinal studies of undernutrition impacts	Fetal malnutrition hypomethylates PCK1 promoter in liver, increasing gluconeogenesis; tissue-specific changes (e.g., GR in hypothalamus); linked to cortisol and metabolic programming.	High similarity to humans in histone/DNA mods but differs from rodents in lifespan/epigenetic stability; limitations: ethical restrictions; high cost; fewer studies on micronutrients; potential confounding by social factors.
Zebrafish (*Danio rerio*)	Rapid development; transparent embryos; easy diet manipulation in water; high fecundity for large sample sizes; genetic manipulation (e.g., CRISPR) feasible	Validation of interventions in a highly translatable model (e.g., supplement trials before human studies) Micronutrient deficiencies and developmental biology (e.g., vitamin D, E effects on bone and muscle) High-throughput screening of diets or compounds that ameliorate malnutrition Nutrient gene interaction (nutrigenomics) and metabolic regulation studies	Micronutrient deficiency distorts liver DNA methylation (e.g., in one-carbon genes); plant diets cause DMCs in immunity/MAPK genes; affects stress response and homeostasis.	Similar to mammals in DNA methylation but lacks histone mods detail; differs in aquatic nutrition/epigenetic resets during embryogenesis; limitations: non-mammalian; short-term focus; less relevant for gut microbiota effects.
Guinea Pig (*Cavia porcellus*)	Requires dietary vitamin C (like humans); similar pregnancy physiology (hemochorial placenta); docile medium-sized rodent	Modeling obesity and liver disease with high-fat/high-glucose diets Scurvy (vitamin C deficiency) studies and antioxidant research Perinatal undernutrition impacts (offspring development under maternal deficiency) Immunological changes under protein or micronutrient malnutrition (some immune aspects more similar to human than mouse)	Vitamin C deficiency impairs DNA demethylation via TET enzymes; affects neurogenesis/hippocampal genes; indirect histone mods via oxidative stress.	Similar to humans/primates in Vit C dependency (differs from rodents); limitations: limited epigenetic data; focus on scurvy; potential sex-specific effects unstudied.
Chicken (*Gallus gallus*)	Rapid growth rate; egg embryo allows nutrient manipulation in ovo; well-studied in agriculture; different phylogenetic perspective (avian)	Histopathology of chronic malnutrition (connective tissue, bone changes in deficiency) Historical discovery of vitamins (e.g., thiamine/B₁ deficiency causing polyneuritis) Protein malnutrition and growth stunting in chicks (modeling kwashiorkor marasmus in simplified form) Bone and cartilage development under mineral deficiencies (e.g., rickets studies) Testing of diet supplements and feed efficiency (indirectly informing human nutrition) Investigation of fatty liver and protein metabolism in oviparous model	Prenatal protein undernutrition alters hepatic transcriptome via DNA methylation (e.g., hypomethylation in lipid genes); zinc/betaine supplements cause hypomethylation in anti-inflammatory genes.	Similar to mammals in methylation responses to deficiencies but avian-specific (e.g., in ovo manipulation); differs in no placenta; limitations: agricultural bias; fewer transgenerational studies; less cognitive relevance.
Dog (*zebra*)	Omnivore with moderate metabolic similarity to humans; larger body for surgical interventions; historical model for certain human deficiencies	Historical pellagra model: “Black tongue” disease in dogs to identify niacin (vitamin B₃) sources Early protein and Nutrient gene interaction energy metabolism experiments (establishing protein requirements and effects of starvation) Current use: Limited in malnutrition research (occasionally used for advanced testing of therapeutic foods or in comparative obesity studies)	Niacin deficiency affects NAD levels, indirectly impacting sirtuins for histone deacetylation; potential DNA repair via PARPs; no direct studies, but inferred from NAD-epigenome links.	Similar to mammals in NAD-dependent mods but limited data (differs from rodents in niacin metabolism efficiency); limitations: historical/outdated; ethical/cost issues; poor for modern epigenetics due to lack of genetic tools.

Each model organism above has shed light on different facets of malnutrition. Additionally, Table 3 provides a comparative summary of animal models. Ratings are based on established biomedical literature, reflecting relative assessments (e.g., cost considers housing, breeding, and ethical factors; physiological similarity emphasizes metabolic and digestive parallels to humans) (26, 75–79). By leveraging the unique features of each species, researchers can investigate malnutrition at levels ranging from genes and molecules (mice, zebrafish) to whole-organism physiology and behavior (pigs, primates). The primary applications listed show how each species contributes i-e rodents and zebrafish often uncover mechanisms and potential interventions, whereas pigs and primates strengthen translational relevance. It is through a combination of these models, aligned with ethical use and guided by the 3Rs, that science advances our understanding of malnutrition and informs strategies to alleviate it. Ultimately, findings from animal models must be integrated with human studies to fully bridge the translational gap, but animal research remains a cornerstone for unraveling the complex biology of malnutrition.

**Table 3:** Comparative overview of animal models in malnutrition research.

Animal model	Cost	Physiological similarity to humans	Genetic manipulability	Translational relevance
Mouse	Low (small size, rapid breeding reduces expenses)	Medium (conserved metabolic pathways but differences in liver function and energy use)	High (extensive CRISPR and knockout tools for gene-diet interactions)	High (mechanistic insights into micronutrient deficiencies and obesity, though scaling to humans requires caution)
Rat	Low (affordable maintenance, similar to mice)	Medium (good for neurobehavioral and developmental programming, but islet differences affect diabetes models)	High (inbred strains and transgenics for metabolic syndrome)	High (strong for prenatal undernutrition effects on later health, with direct parallels to human epidemiology)
Pig	High (larger facilities and longer lifespans increase costs)	High (omnivorous digestion, gut microbiota, and organ size closely mimic humans)	Medium (emerging transgenics, but less routine than rodents)	High (excellent for gut-nutrition interactions and pediatric malnutrition, bridging rodents to clinical trials)
Non-human Primate	Very high (ethical oversight and specialized care elevate expenses)	Very high (genetic proximity enables life-course studies of diet impacts)	Low (limited due to ethics, focus on natural models)	Very high (gold standard for fetal programming and cognitive effects of undernutrition)
Zebrafish	Low (high fecundity, simple aquatic housing)	Low-medium (useful for early development and micronutrient effects, but non-mammalian limits)	High (CRISPR for nutrigenomics and rapid screening)	Medium (valuable for high-throughput deficiency studies, less for complex human metabolism)
Guinea Pig	Medium (specific diet needs like vitamin C add modest costs)	Medium-high (shares scurvy susceptibility and placental traits with humans)	Low-medium (limited tools, strain-based studies)	Medium (strong for vitamin deficiencies and perinatal undernutrition)
Chicken	Low (agricultural scalability, in ovo manipulation)	Low (avian metabolism differs, no placenta for maternal effects)	Medium (embryo genetic edits possible)	Medium (useful for vitamin discoveries and growth stunting, but phylogenetic distance reduces applicability)
Dog	High (larger size, veterinary care requirements)	Medium-high (omnivore traits align with human nutrient metabolism)	Low (focus on natural breeds, rare engineering)	Medium (historical for deficiencies like pellagra, now limited but relevant for comparative obesity)

Animal models are evaluated across key criteria (cost, physiological similarity to humans, genetic manipulability, and translational relevance), with ratings assigned as low, medium, or high based on their relative performance in nutrition-related studies.

## 5 Future perspective and emerging trends

The use of animal models in malnutrition research is evolving rapidly. With growing advances in biotechnology, molecular biology, and systems science, new frontiers are emerging that promise to significantly enhance the relevance, precision, and translational value of animal studies in nutrition research. Despite limited recent literature directly addressing emerging trends under this specific framing, current research is converging on several key innovations. Genetically modified rodents and gnotobiotic animals, particularly germ-free mice colonized with human microbiota, are being increasingly employed to dissect host-microbiome interactions that influence nutrient absorption and immune regulation in malnourished states (80). Humanized gnotobiotic mouse models, where germ-free mice are colonized with human gut microbiota have revolutionized our understanding of host-microbiota interactions in the context of malnutrition (81). These models are instrumental in studying how microbial communities influence nutrient absorption, immune development, and metabolic programming (82). Omics technologies, including transcriptomics, metabolomics, and proteomics, are augmenting mechanistic insights by enabling the study of systemic responses to nutrient deficiencies at cellular and molecular levels.

The integration of multi-omics platforms such as, transcriptomics, proteomics, metabolomics, and epigenomics, with animal models has opened new avenues to map the molecular cascades affected by malnutrition. These high-resolution techniques allow for comprehensive profiling of changes at the cellular and systemic levels in response to nutritional deprivation or excess. Systems biology approaches facilitate the construction of interaction networks, enabling the identification of key regulatory nodes and potential therapeutic targets (83). These integrative strategies not only deepen our mechanistic understanding but also support the development of predictive models for nutritional outcomes. Translating omics results from animal models to clinical practice requires cost- and time-effective strategies to bridge preclinical insights with human applications. For instance, multi-omics profiling in rodent models of undernutrition has identified epigenetic biomarkers (e.g., altered methylation) that predict metabolic risks, which can be validated in human cohorts via affordable targeted sequencing panels rather than whole-genome omics (84). Costs have plummeted with next-generation sequencing (now <$1,000/genome), enabling scalable biomarker discovery; AI-driven data integration further accelerates analysis from months to days (85). In malnutrition, this could inform personalized interventions, like micronutrient supplements tailored to gut microbiomics, tested first in pigs for translational fidelity before low-cost human trials. Challenges include validation across diverse populations, but fit-for-purpose animal models (e.g., humanized microbiomes) enhance efficiency.

One of the most promising advancements is the development of genetically modified animal models, particularly using CRISPR/Cas9 and other genome-editing tools. These models allow researchers to study specific gene-nutrient interactions and unravel the role of genetic susceptibilities in the manifestation of malnutrition-related phenotypes (86). Such precision models enable the dissection of nutrient-specific pathways and their downstream physiological consequences, contributing to the formulation of targeted nutritional interventions.

While not animal models per se, advances in 3D organoid cultures and bio-printing technologies offer powerful adjuncts to *in vivo* studies. These systems, derived from animal or human stem cells, can mimic tissue-specific responses to malnutrition in a controlled environment. Organoids of the intestine, liver, or brain, for instance, allow researchers to study tissue-specific nutrient sensing, absorption, and pathology, complementing whole-animal studies and reducing the reliance on live animals in preliminary investigations (87).

Future directions also prioritize the refinement of ethical and translational considerations, advocating for models that more closely recapitulate human pathophysiology, including the use of non-human primates and 3D organoid systems to complement in vivo findings. Collectively, these innovations promise to deepen our mechanistic understanding of malnutrition, guide biomarker discovery, and inform therapeutic and policy interventions for diverse populations.
